# Age at Onset Impact on Clinical Profile, Treatment, and Real-Life Perception in Spondyloarthritis Patients, Enhancing a Personalized Approach: A Monocentric Cohort Analysis

**DOI:** 10.3390/jpm16020063

**Published:** 2026-01-28

**Authors:** Federico Fattorini, Linda Carli, Cosimo Cigolini, Lorenzo Esti, Marco Di Battista, Marta Mosca, Andrea Delle Sedie

**Affiliations:** 1 Rheumatology Unit, Department of Clinical and Experimental Medicine, University of Pisa, 56126 Pisa, Italy; federicofattorini19@gmail.com (F.F.); lorenzo.esti@gmail.com (L.E.); dibattista.marco91@gmail.com (M.D.B.); marta.mosca@unipi.it (M.M.); 2 Rheumatology Unit, Azienda Ospedaliero Universitaria Pisana (AOUP), 56126 Pisa, Italy; 81clinda@gmail.com (L.C.); cosimo.cigolini@gmail.com (C.C.)

**Keywords:** spondyloarthritis, age at onset, quality of life

## Abstract

**Background**: Spondyloarthritis (SpA) typically develops before 40 years of age, but increasing life expectancy has led to a growing number of cases in older adults. It is well known that age at onset may influence disease presentation, comorbidities, and patient outcomes. **Objectives**: To assess whether age at onset influences SpA clinical presentation. **Methods**: We analyzed clinical, demographic, clinimetric, and imaging data in 272 SpA patients, grouped by onset age: early (≤40, n = 119), intermediate (41–59, n = 127), and late (≥60, n = 26). All patients had a minimum follow-up duration of 12 months. Their epidemiologic, clinic, and clinimetric data were collected, as well as patient-reported outcome measures (PROs) [Patient Global Assessment (PGA), Health Assessment Questionnaire (HAQ), FACIT-Fatigue (FACIT-F), SHORT-FORM 36 (SF-36), Hospital Anxiety and Depression Scale (HADS), Work Productivity and Activity Impairment Questionnaire (WPAI), CSI (Central Sensitization Inventory), and Psoriatic Arthritis Impact of Disease (PsAID) questionnaire]. In univariate analyses, differences in categorical variables across onset groups were assessed using Fisher’s exact test; for continuous variables, between-group comparisons were performed using the Mann–Whitney U test (two-tailed) or the Kruskal–Wallis test, as appropriate, with Bonferroni correction for post hoc analyses. Multivariable regression models were subsequently fitted, adjusting for sex, diagnosis, and disease duration. For binary outcomes, multivariable logistic regression models were used, while multivariable linear regression models (ANCOVA) were applied for continuous outcomes. The overall association between onset group and each outcome was formally tested using likelihood ratio tests, comparing models including the onset variable with nested models excluding it. A *p*-value < 0.05 was considered statistically significant. **Results**: Patients’ mean age was 60.0 ± 13.7 years; 55.9% of them were males; and there were 188 cases (69.1%) of psoriatic arthritis (PsA) and 84 cases (30.9%) of ankylosing spondylitis (AS). In early-onset patients, inflammatory back pain (IBP) was more frequent, whereas late-onset patients more often presented with joint swelling. A family history of SpA and psoriasis was less common in late-onset forms. Comorbidities, including osteoporosis, osteoarthritis, hypertension, hyperuricemia, and diabetes, were more prevalent in older-onset patients, resulting in a higher overall comorbidity burden in Groups 2 and 3. Patient-reported outcomes were largely similar across age groups, although work activity limitation was more pronounced in younger patients. **Conclusions**: Age at onset seems to influence SpA phenotypes: early-onset could favor axial involvement, while late-onset may associate with peripheral arthritis. Late-onset forms are associated with a more severe comorbidity burden, in particular for cardiovascular risk factors. Lung involvement proved to be more prevalent with respect to the general population, so it should be checked in the routinary assessment of SpA patients. These findings suggest that rheumatologists could tailor their routine assessments based on patients’ age at disease onset. Interestingly, work productivity seems more impacted in early-onset patients. All these points highlight the importance of age at disease onset in SpA, guiding toward personalized medicine in terms of follow-up, therapy, and more holistic patient management.

## 1. Introduction

Spondyloarthritis (SpA) is a group of several related but phenotypically distinct disorders: psoriatic arthritis (PsA), arthritis related to inflammatory bowel disease (SpA-IBD), reactive arthritis (ReA), ankylosing spondylitis (AS), and undifferentiated SpA (uSpA) [1].

Despite some differences among its various forms, patients are typically seronegative, lacking rheumatoid factor (RF) in the serum, and often present with inflammatory back pain due to sacroiliac joint inflammation. Peripheral arthritis is usually asymmetric, predominantly affecting the lower limbs and large joints. Moreover, both peripheral and axial enthesitis are some of the most typical involvements. Extra-articular manifestations are common and may include ocular (uveitis), cutaneous (psoriasis), and gastrointestinal involvement (IBD) [2].

The current trend is to move beyond the previous nosographic classification. In 2012, Braun et al. proposed a distinction of SpA into predominantly axial forms (axSpA), primarily affecting the spine and presenting with spondylitis and sacroiliitis, and peripheral forms (pSpA), which are characterized by arthritis, peripheral enthesitis, and dactylitis [3,4].

The Assessment of SpondyloArthritis International Society (ASAS) has played a pivotal role in the development of contemporary classification criteria for SpA through the conduct of multiple international clinical studies. These efforts culminated in the evolution of the original 1984 modified New York criteria for ankylosing spondylitis (AS) [5] to the 2009 ASAS classification criteria [6].

A key innovation of the ASAS criteria was the incorporation of magnetic resonance imaging (MRI) in addition to conventional radiography, thereby enabling earlier detection of inflammatory changes and improving diagnostic accuracy.

According to the ASAS classification criteria, patients presenting with chronic back pain lasting at least three months and with symptom onset before the age of 45 years may be classified as having axSpA if imaging evidence of sacroiliitis is demonstrated on pelvic radiographs or MRI, in conjunction with at least one additional clinical feature of SpA.

Similarly, the ASAS criteria for pSpA allow for classification in patients presenting with peripheral arthritis, enthesitis, or dactylitis, provided that these manifestations are accompanied by one or more additional SpA features.

These criteria were validated in patients under 45 years of age, as axSpA usually occurs in the second or third decade of life, with less than 5% of patients diagnosed when older. However, all forms of SpA may present with a late onset, thus creating a persistent diagnostic and therapeutic challenge [7]. In particular, the prevalence of the whole group of late-onset SpA has been estimated to range between 3 and 8% [8], with a growing numerical prevalence due to increase life expectancy [9].

In older patients, diagnosis is often complicated by the coexistence of multiple musculoskeletal conditions that can obscure the underlying disease. Consequently, late-onset SpA is frequently misdiagnosed as rheumatoid arthritis, polymyalgia rheumatica, crystal-induced arthritis, or osteoarthritis [9].

One of the primary challenges in evaluating late-onset SpA is the absence of a globally accepted age threshold (whether 45 [10,11], 50 [12], or 60 years [13]). This lack of consensus leads to heterogeneous patient clusters across studies, often yielding confounding results. While some literature suggests that an older age at onset is associated with distinct clinical presentations, other researchers, such as Olivieri et al., have observed that onset age does not significantly alter clinical features or prognosis [7]. This suggests that early-onset and late-onset SpA should perhaps not be categorized as distinct clinical entities.

In light of these considerations, there is a clear need to validate the ASAS criteria for axial and peripheral SpA in patients with axial disease or undifferentiated SpA whose symptoms begin after the age of 45 in order to improve diagnostic accuracy and clinical management [14].

Considering these diagnostic uncertainties and the limited data on age-related differences in SpA, elucidating the influence of disease onset timing on clinical phenotype and patient-reported experience remains a critical unmet need. Starting from this assumption, we aimed at evaluating whether the age at onset could influence the clinical presentation, disease course, or QoL in a cohort of patients with SpA regularly followed in our outpatient clinic.

## 2. Materials and Methods

### 2.1. Study Design and Population

This cross-sectional study included consecutive adult outpatients evaluated at the SpA Clinic of the Rheumatology Unit of Pisa. All patients had a diagnosis of psoriatic arthritis (PsA) or ankylosing spondylitis (AS) and met the ClASsification criteria for Psoriatic Arthritis (CASPAR) or Assessment of SpondyloArthritis international Society (ASAS) classification criteria, respectively. Patients were regularly followed at the SpA outpatient clinic and consecutively enrolled between November 2021 and May 2023. Age at onset was defined as the age at which patients first experienced SpA-related musculoskeletal symptoms, as reported by the patient and confirmed through medical records when available. Clinical, demographic, clinimetric, and imaging parameters were compared among the groups with early (≤40 years) [Group 1], intermediate (>40 and < 60 years) [Group 2], and late onset (≥ 60 years) [Group 3] at a cross-sectional level. Although patients were followed longitudinally in routine clinical practice for at least 12 months, the analyses presented in this manuscript are strictly cross-sectional and based on data collected at a single time point. Follow-up duration was not used to derive longitudinal outcomes and did not influence the statistical analyses.

### 2.2. Data Collection

The following epidemiologic, clinical, and clinimetric data were collected: age, sex, diagnosis, age at onset, symptoms at onset, extra-articular manifestations (psoriasis, uveitis, or IBD), joint involvement (axial or peripheral arthritis), enthesitis, and dactylitis. Disease activity was assessed using the Ankylosing Spondylitis Disease Activity Score with C-reactive protein (ASDAS-CRP) and the Disease Activity in PSoriatic Arthritis (DAPSA) score.

We recorded the occurrence of the following comorbidities: osteoporosis (OP), defined by the presence of a bone mineral density T-score ≤ −2.5 at the lumbar spine, femoral neck, or total hip measured by dual-energy X-ray absorptiometry (DXA), according to World Health Organization criteria and/or current treatment with anti-osteoporotic medication [15]); symptomatic osteoarthritis (OA), supported by typical clinical and/or radiographic features; arterial hypertension (AH); chronic obstructive pulmonary disease (COPD); interstitial lung disease (ILD); ischemic heart disease; fibromyalgia (FM), defined according to the 2016 American College of Rheumatology (ACR) diagnostic criteria [16]; diabetes; hyperuricemia (HU), defined by serum uric acid levels > 7.0 mg/dL in males or >6.0 mg/dL in females and/or current urate-lowering therapy; thyroid disorders (history of clinically diagnosed thyroid disease, including hypothyroidism or hyperthyroidism, requiring medical treatment or ongoing follow-up); dyslipidaemia (according to values of total cholesterol > 200 mg/dL and/or LDL > 116 mg/dL, and/or HDL < 40 mg/dL in males or <50 mg/dL in females, and/or triglycerides > 150 mg/dL); obesity [defined as a body mass index (BMI) ≥ 30 kg/m^2^]; and psychiatric disorders (defined as a history of clinically diagnosed anxiety, depression, or other psychiatric conditions requiring pharmacological treatment or specialist follow-up). Patients who were minors or did not provide informed consent were excluded.

### 2.3. Treatment History

A complete pharmacological history was collected, including the use of non-steroidal anti-inflammatory drugs (NSAIDs), glucocorticoids (GCs), conventional synthetic disease-modifying antirheumatic drugs (csDMARDs), biological DMARDs (bDMARDs), and targeted synthetic DMARDs (tsDMARDs).

### 2.4. Patient-Reported Outcomes (PROs)

The following PROs were administered: Patient Global Assessment (PGA), Health Assessment Questionnaire (HAQ), Functional Assessment of Chronic Illness Therapy–Fatigue (FACIT-F), Short Form 36 (SF-36), Hospital Anxiety and Depression Scale (HADS), Work Productivity and Activity Impairment Questionnaire (WPAI), CSI (Central Sensitization Inventory), and Psoriatic Arthritis Impact of Disease (PsAID) questionnaire.

### 2.5. Statistical Analysis

Categorical variables were reported as absolute and relative frequencies, while continuous data were described using mean and standard deviation. In univariate analyses, differences in categorical variables across onset groups were assessed using Fisher’s exact test. For continuous variables, given that most of them represented clinical scores or were not normally distributed, between-group comparisons were initially performed using the Mann–Whitney U test (two-tailed) or the Kruskal–Wallis test, as appropriate, with Bonferroni correction for post hoc analyses.

To evaluate whether the observed associations between onset group and the variables of interest were independent of potential confounders, multivariable regression models were subsequently fitted, adjusting for sex, diagnosis, and disease duration. For binary outcomes, multivariable logistic regression models were used, with the variable of interest entered as the dependent variable and onset group as the main independent factor. For continuous outcomes, multivariable linear regression models (ANCOVA) were applied using the same set of covariates.

The overall association between onset group and each outcome was formally tested using likelihood ratio tests, comparing models including the onset variable with nested models excluding it. Only variables showing a statistically significant overall association with onset were considered relevant and reported in the main results tables.

When the overall test was significant, post hoc pairwise comparisons between onset groups were performed based on the fitted multivariable models, using estimated marginal means. Bonferroni correction was applied to account for multiple testing. All post hoc analyses were adjusted for sex, diagnosis, and disease duration. Significance was set at 0.05 and all analyses were carried out with R software (R Core Team 2025, R 4.5.0 Foundation for Statistical Computing, Vienna, Austria).

### 2.6. Ethical Considerations

This study was conducted in accordance with the Declaration of Helsinki and received approval from the local ethics committee (Comitato Etico di Area Vasta Nord Ovest, reference number 20070; 9 September 2021). Written informed consent for participation was obtained from all patients.

## 3. Results

A total of 272 patients were enrolled, 152 male (55.9%), with a mean age of 60.0 ± 13.7 years; 188 patients (69.1%) had a diagnosis of PsA and 84 (30.9%) of AS. Table 1 summarized the demographic and clinical characteristics of the study population.

### 3.1. Correlations Among Epidemiological Parameters

Correlations among epidemiological parameters, including family history, in the three study groups are summarized in Table 2.

Gender differed significantly among the three groups (*p* = 0.01); post hoc analysis showed that female gender was significantly more frequent in Group 2 compared to Group 1 (*p* = 0.009). In late-onset SpA, the weight of a family history for SpA and psoriasis (*p* = 0.05 between Group 1 and 2) appears less significant when compared with younger onset.

### 3.2. Comorbidities

Comorbidity burden differed significantly across groups (Table 3), increasing from Group 1 to Group 3, and the proportion of patients with ≥3 comorbidities was highest in Group 3 (61.5%), followed by Group 2 and Group 1 (all *p* < 0.01).

OP and OA were more frequent in Group 3 vs. Group 1 (both *p* < 0.001) and, for OA, also vs. Group 2 (*p* = 0.003). AH was more prevalent in Group 3 and Group 2 vs. Group 1 (*p* = 0.001 and *p* = 0.003, respectively). ILD was more common in Group 2 vs. Group 1 (*p* = 0.004), whereas COPD was more frequent in Group 3 vs. Group 1 and Group 2 (*p* = 0.01 and *p* = 0.05).

Metabolic comorbidities were increased in Group 3, with higher prevalence of HU and DM compared with Group 1 and Group 2 (both *p* < 0.001).

### 3.3. Clinical Features and Disease Onset

Correlations between clinical features and disease onset characteristics are shown in Table 4.

### 3.4. Medication Use

Analysis of medication use across age-at-onset groups revealed differences in treatment patterns (Table 5). In fact, methotrexate was increasingly prescribed with advancing age at onset, showing the highest frequency in late-onset patients, with a significant difference between Group 3 and Group 1.

### 3.5. Clinimetric Measures and Age-at-Onset Groups

The main clinimetric measures across the three age-at-onset groups are summarized in Table 6, showing overall comparable disease activity scores, except for a significantly higher swollen joint count in late-onset patients.

### 3.6. PROs and Age-at-Onset Groups

Across the groups, most PROs—including HAQ, FACIT, SF-36 subscales, HADS, CSI, and PSAID—showed no statistically significant differences.

We noted that work-related functioning, as measured by the WPAI work activity limitation domain, showed a significant difference between younger and late-onset patients (*p* = 0.03), with early-onset individuals experiencing greater limitations in work productivity.

## 4. Discussion

This study provides a comprehensive overview of a monocentric cohort of patients with SpA stratified by age at disease onset, with particular attention to clinical presentation, comorbidity profile, and PROs. While previous studies have often focused on specific SpA subtypes or younger populations, our work captures both early-, intermediate-, and late-onset patients, allowing for a comparison across different age groups.

By integrating demographic, clinical, imaging, and PRO data, this study characterizes the phenotypic variations associated with age at onset, also trying to highlight how disease burden and QoL could be differentially affected across the lifespan. Importantly, the inclusion of PROs such as SF-36 domains, HAQ, FACIT-F, and WPAI provides a nuanced understanding of how both younger and older patients perceive the impact of their disease on daily functioning, emotional well-being, and work productivity.

This study allows us to identify age-specific patterns, such as the predominance of axial involvement in early-onset patients versus peripheral arthritis and higher comorbidity burden in late-onset patients, which can inform tailored monitoring, therapeutic strategies, and resource allocation.

The prevalence of late-onset forms in our cohort was 9%, similar to what was already observed in a Turkish cohort [17].

Consistently with two previous Spanish studies [18,19], in early-onset forms we confirmed the weight of family history and genetic background; moreover, we observed a higher prevalence of axial involvement. On the contrary, also in agreement with a study from Japan, the late-onset subgroup showed a higher prevalence of peripheral involvement [20], with a numerically higher prevalence of clinical and ultrasound parameters and a significant difference in terms of swollen joints. These results agree also with a large Brazilian cohort of SpA patients [21].

Interestingly, we did not observe a major impact of extra-axial manifestations of SpA among younger patients; these data do not align with data from Kishimoto et al., who found a higher prevalence of both hip and knee involvement in early-onset cases [22].

We observed a higher cardiovascular comorbidity burden in late-onset cases (AH, diabetes, and HU), as also already highlighted in Spanish patients [18,19], even if the prevalence of dyslipidemia or obesity did not significantly differ among subgroups. Interestingly, the prevalence of COPD in our cohort of SpA patients is similar of the one registered in the general population, with a significantly higher prevalence in older age onset. These data are similar to those already described by Monosi et al. [23]. Concordantly, our patients in Group 3 presented a significantly higher number of swollen joints, which was considered as a major factor risk for COPD in PsA patients [23]. A possible pathophysiologic link between PsA and COPD has been hypothesized [24]. Anyway, this data remain uncertain as the prevalence of COPD was not increased in patients with SpA compared to the general population in a Swedish study [25]

Considering ILD in our cohort, we noticed a similar (Group 3) or even higher (Group 2) prevalence with respect to Monosi et al. [23], where, again, swollen joint count was demonstrated to be the major risk factor in their study. In our population we had a higher prevalence in Group 2 (significant) and 3 (numerical) with respect to Group 1. In accordance with this result, we previously demonstrated a significantly higher prevalence of ILD in SpA patients when using US to demonstrate irregularity of the pleural line [26].

No differences emerged among the various groups with respect to disease activity parameters, in line with the findings of Brophy et al. [27].

Age at onset seems to influence not only the clinical picture, but also QoL outcomes and work productivity.

Of note, younger patients seem to experience a greater limitation in their work ability, thus confirming how SpA could worsen patients’ social functioning, especially at the height of their productive life. On the contrary, elderly patients reported a greater subjective burden of disease, evaluated by PGA.

Our results show that identifying early-, intermediate-, and late-onset patients could help in planning different individualized management strategies, thus permitting the prevention of the development of potential comorbidities, to optimize their management and to maximize therapeutic responses, aiming at avoiding a “one-size-fits-all” approach. Interestingly, conditions such as FM and OA could lead to an overestimation of disease activity, potentially resulting in an escalation in SpA therapy instead of targeted treatment for comorbidities.

Overall, this study offers a holistic perspective, potentially bridging the gap between epidemiological data and patient outcomes. It underscores the importance of considering age at onset as a key determinant of SpA phenotype, comorbidity risk, and patients’ functioning, thus providing a foundation for personalized patient management and age-adapted care pathways.

This study has several limitations. First, its monocentric and cross-sectional design limits the generalizability of our findings and precludes causal inferences regarding the relationship between age at onset and disease outcomes. Second, the study population included different SpA entities (PsA and AS), which are clinically different. While this reflects real-world clinical practice, it introduces heterogeneity that may confound age-related comparisons. Stratified or multivariable analyses adjusting for diagnosis, sex, and treatment exposure were limited by the relatively small size of the late-onset subgroup and may have been underpowered. We suppose that, by enrolling a higher number of patients in Group 3, most of the numerically superior prevalence shown in Group 3 could become significant. Additionally, disease-specific instruments such as PSAID and ASDAS were applied within a heterogeneous cohort, which may limit interpretability despite their use according to validated indications. Finally, the relatively small number of late-onset patients and the absence of systematic imaging-based analyses further limit the robustness of subgroup comparisons. Future multicentre and longitudinal studies are needed to confirm these findings and better define the role of age at disease onset in SpA.

## 5. Conclusions

It is essential to recognize that age at disease onset in SpA is not simply a descriptive feature, but a factor with significant implications for disease progression and patient management.

In particular, axial involvement was more frequent in patients with an earlier onset, whereas peripheral arthritis (swollen joints) was more frequent in the elderly. As expected, our patients belonging to Group 3 showed a higher risk of comorbidities, in particular cardiovascular risk factors. Based on our preliminary results, the routinary assessment of SpA patients could be focused on screening for lung diseases, as we confirmed COPD and ILD as comorbidities more prevalent with respect to the general population. In our study ILD was significantly more present in Group 2, so lung evaluation should especially be performed in SpA patients older than 40 years old.

No significant differences emerged in QoL. Interestingly, patients with an early onset showed a higher impairment in work productivity.

These results could help in phenotyping SpA patients and highlight the opportunity to not underestimate the senile-onset forms of SpA that seem associated with a higher cardiovascular risk; moreover, our data underline how young patients, in the prime of their working life, could suffer from the disease burden.

A personalized medicine approach, based on these results, could permit improvement in patients’ outcomes, leading to an optimization of resources.

## Figures and Tables

**Table 1:** Demographic and clinical characteristics.

Characteristic	Value
Gender, n (%)	F: 120 (44.1), M: 152 (55.9)
Mean Age, mean SD), years	60.0 (13.7)
Diagnosis, n (%)	PsA: 188 (69.1), AS: 84 (30.9)
Age at Onset, mean SD), years	40.9 (13.9)
Onset Classification, n (%)	Early: 119 (43.8), Intermediate: 127 (46.7), Late: 26 (9.6)

PsA: psoriatic arthritis; AS: ankylosing spondylitis.

**Table 2:** Correlations with epidemiological data and family history.

Characteristic	Group 1, n = 119	Group 2, n = 127	Group 3, n = 26	*p*-Value (Overall)
Female sex, n (%)	41 (34.5)	68 (53.5)	11 (42.3)	0.01 group 2 vs. 1
BMI, mean (SD), kg/m^2^	26.5 (4.8)	28.2 (7.7)	27.2 (3.2)	NS
Smoking habit, n (%)	12 (10.1)	25 (19.7)	5 (19.2)	NS
Disease duration, mean (SD), years	23.8 (12.8)	16.6 (9.7)	10.9 (6.0)	<0.001 group 1 vs. 2 and 3; 0.003 group 2 vs. 3
Diagnosis of AS, n (%)	57.0 (47.9)	25.0 (19.7)	2.0 (7.7)	<0.001 group 1 vs. 2 and 3
Diagnosis of PsA, n (%)	62.0 (52.1)	102.0 (80.3)	24.0 (92.3)	<0.001 group 2 and 3 vs. 1
Family history of SpA, n (%)	9 (7.5)	2 (1.5)	0 (0.0)	0.05 group 1 vs. 2
Family history of psoriasis, n (%)	8 (6.7)	20 (15.7)	0 (0.0)	0.05 group 2 vs. 3

BMI: body mass index; AS: ankylosing spondylitis; PsA: psoriatic arthritis; SpA: spondyloarthritis; NS = not significant.

**Table 3:** Correlations with comorbidities.

Comorbidities	Group 1, n = 119	Group 2, n = 127	Group 3, n = 26	*p*-Value-Adjusted
OP, n (%)	7 (5.9)	17 (13.3)	6 (23.1)	<0.001 Group 3 vs. 1 and 0.02 Group 2 vs. 1
OA, n (%)	23 (19.3)	41 (32.3)	16 (61.5)	<0.001 Group 3 vs. 1 and 0.003 Group 3 vs. 2
Cardiovascular disease, n (%)	15 (12.6)	22 (17.3)	5 (19.2)	NS
AH, n (%)	25 (21.0)	42 (33.0)	11 (42.3)	0.001 Group 3 vs. 1 and 0.003 Group 2 vs. 1
Kidney disease, n (%)	7 (5.8)	12 (9.4)	3 (11.5)	NS
ILD, n (%)	3 (2.5)	15 (11.8)	2 (7.7)	0.004 Group 2 vs. 1
COPD, n (%)	7 (5.8)	7 (5.5)	4 (15.4)	0.01 Group 3 vs. 1 and 0.05 Group 3 vs. 2
Eye involvement, n (%)	10 (8.4)	9 (7.0)	1 (3.8)	NS
Psychiatric disease, n (%)	13 (10.9)	8 (6.3)	1 (3.8)	NS
TDs, n (%)	13 (10.9)	32 (25.2)	3 (11.5)	NS
Obesity, n (%)	11 (9.2)	19 (14.9)	2 (7.7)	NS
HU, n (%)	9 (7.5)	11 (8.6)	9 (34.6)	0.001 Group 3 vs. 1 and 2
Diabetes, n (%)	9 (7.5)	11 (8.6)	9 (34.6)	<0.001 Group 3 vs. 1 and 2
FM, n (%)	14 (11.7)	32 (25.2)	1 (3.8)	NS
Dyslipidemia, n (%)	20 (16.8)	27 (21.2)	9 (34.6)	NS
Total comorbidities, mean (SD)	1.4 (1.7)	2.2 (1.8)	3.3 (1.9)	<0.001 Group 3 vs. 1 and Group 2 vs. 1; 0.001 Group 3 vs. 2
Comorbidities ≥ 3, n (%)	26 (21.8)	42 (33.0)	16 (61.5)	<0.001 Group 3 vs. 1; 0.001 Group 3 vs. 2; 0.01 Group 2 vs. 1

OP: osteoporosis; OA: osteoarthritis; AH: arterial hypertension; ILD: interstitial lung disease; COPD: chronic obstructive pulmonary disease; TD: thyroid disease; HU: hyperuricemia; FM: fibromyalgia; NS = not significant.

**Table 4:** Correlation with clinical characteristics of disease onset.

Characteristics at Disease Onset Mean (SD)	Group 1, n = 119	Group 2, n = 127	Group 3, n = 26	*p*-Value-Adjusted
Clinical arthritis	68 (57.1)	87 (68.5)	22 (84.6)	NS
Clinical dactylitis	15 (12.6)	14 (11.0)	4 (15.3)	NS
Clinical enthesitis	24 (20.1)	35 (27.5)	2 (7.7)	NS
Clinical tenosynovitis	16 (13.4)	27 (21.2)	4 (15.3)	NS
Ultrasound synovitis	35 (29.4)	44 (34.6)	8 (30.7)	NS
Ultrasound dactylitis	1 (0.8)	2 (1.5)	1 (3.8)	NS
Ultrasound enthesitis	2 (1.6)	6 (4.7)	2 (7.7)	NS
Ultrasound tenosynovitis	6 (5.0)	17 (13.3)	3 (11.5)	NS
Erosions	4 (3.3)	7 (5.5)	2 (7.7)	NS
IBP	25 (20.5)	14 (10.8)	2 (7.1)	0.046 (Group 1 vs. 3)
Syndesmophytes	3 (2.5)	4 (3.1)	0 (0.0)	NS
Sacroiliitis	21 (17.6)	21 (16.5)	3 (11.5)	NS
Spondylitis	10 (8.4)	7 (5.5)	1 (3.8)	NS

IBP: inflammatory back pain; NS = not significant.

**Table 5:** Correlations with medications used in history, including csDMARDs, tsDMARDs, and bDMARDs.

Characteristic Mean (SD)	Group 1, n = 119	Group 2, n = 127	Group 3, n = 26	*p*-Value-Adjusted
MTX	67 (56.3)	91 (71.6)	23 (88.4)	0.04 group 3 vs. 1
LEF	22 (18.4)	25 (19.6)	8 (30.7)	NS
SZP	39 (32.8)	33 (25.4)	5 (21.4)	NS
HCQ	20 (16.8)	37 (29.1)	4 (15.3)	NS
CyA	23 (19.3)	12 (9.4)	5 (19.2)	NS
Apremilast	3 (0)	10 (3.8)	3 (7.1)	NS
JAKi	4 (2.5)	4 (3.1)	3 (11.5)	NS
TNF-α inhibitors	89 (74.8)	73 (57.4)	17 (65.3)	NS
IL-17 inhibitors	28 (23.5)	27 (21.2)	4 (15.3)	NS
IL-23 inhibitors	8 (6.7)	9 (7.0)	1 (3.8)	NS

SD: standard deviation; MTX: methotrexate; LEF: leflunomide; SZP: sulfasalazine; HCQ: hydroxychloroquine; CyA: cyclosporine A; JAKi: Janus kinase inhibitors; TNF-α: tumor necrosis factor alpha; IL: interleukin; NS = not significant.

**Table 6:** Clinimetric characteristics across the three age-at-onset groups. ASDAs-CRP was used only in AS patients; DAPSA was used only in PsA patients.

Clinimetric Value Mean (SD)	Group 1, n = 119	Group 2, n = 127	Group 3, n = 26	*p*-Value-Adjusted
ASDAS-CRP	1.7 (1.0)	2.1 (1.0)	0.7 (NA)	NS
DAPSA	8.8 (5.7)	10.4 (6.7)	7.0 (6.0)	NS
MASES	0.2 (0.7)	0.4 (1.2)	0.2 (0.7)	NS
LEI	0.1 (0.4)	0.3 (0.7)	0.2 (0.7)	NS
SPARCC	0.3 (0.8)	0.7 (1.4)	0.3 (0.7)	NS
PGA	3.3 (2.5)	4.3 (2.5)	3.9 (3.0)	NS
TJ	0.3 (1.0)	1.2 (2.9)	1.6 (3.1)	NS
SJ	0.2 (0.7)	0.1 (0.3)	1.5 (2.7)	<0.001 group 3 vs. 1 and 2
VAS pain	3.5 (2.8)	4.0 (2.5)	2.9 (2.3)	NS

SD: standard deviation; ASDAS-CRP: ankylosing spondylitis disease activity score with c-reactive protein; DAPSA: disease activity in psoriatic arthritis; MASES: maastricht ankylosing spondylitis enthesitis score; LEI: leeds enthesitis index; SPARCC: spondyloarthritis research consortium of canada score; PGA: patient global assessment; TJ: tender joint; SJ: swollen joint; VAS: visual analogic scale; NA: not available; NS = not significant.

## Data Availability

The raw data supporting the conclusions of this article will be made available by the authors on request.

## Abbreviations

The following abbreviations are used in this manuscript:

SpA	Spondyloarthritis
PsA	Psoriatic Arthritis
axSpA	Axial Spondyloarthritis
pSpA	Peripheral Spondyloarthritis
IBD	Inflammatory Bowel Disease
ReA	Reactive Arthritis
AS	Ankylosing Spondylitis
uSpA	Undifferentiated Spondyloarthritis
RF	Rheumatoid Factor
ASAS	Assessment of SpondyloArthritis International Society
CASPAR	Classification Criteria for Psoriatic Arthritis
AH	Arterial Hypertension
OA	Osteoarthritis
OP	Osteoporosis
COPD	Chronic Obstructive Pulmonary Disease
FM	Fibromyalgia
HU	Hyperuricemia
TD	Thyroid Disease
NSAIDs	Non-Steroidal Anti-Inflammatory Drugs
GCs	Glucocorticoids
csDMARDs	Conventional Synthetic DMARDs
bDMARDs	Biological DMARDs
tsDMARDs	Targeted Synthetic DMARDs
PROs	Patient-Reported Outcomes
PGA	Patient Global Assessment
HAQ	Health Assessment Questionnaire
FACIT-F	Functional Assessment of Chronic Illness Therapy–Fatigue
SF-36	Short Form 36 Health Survey
HADS	Hospital Anxiety and Depression Scale
WPAI	Work Productivity and Activity Impairment Questionnaire
CSI	Central Sensitization Inventory
PsAID	Psoriatic Arthritis Impact of Disease
ASDAS-CRP	Ankylosing Spondylitis Disease Activity Score with CRP
DAPSA	Disease Activity in Psoriatic Arthritis
LEI	Leeds Enthesitis Index
MASES	Maastricht Ankylosing Spondylitis Enthesitis Score
SPARCC	Spondyloarthritis Research Consortium of Canada Score
TJs	Tender Joints
SJs	Swollen Joints
VAS	Visual Analogic Scale
